# Prevalence of Deleterious Oral Habits among 3- to 5-year-old Preschool Children in Bhubaneswar, Odisha, India

**DOI:** 10.5005/jp-journals-10005-1513

**Published:** 2018-06-01

**Authors:** Kanika S Dhull, Tulika Verma, Brahmananda Dutta

**Affiliations:** 1 Reader, Department of Pedodontics and Preventive Dentistry, Kalinga Institute of Dental Sciences, KIIT University, Bhubaneswar Odisha, India; 2 Postgraduate Student, Department of Pedodontics and Preventive Dentistry, Kalinga Institute of Dental Sciences, KIIT University, Bhubaneswar Odisha, India; 3 Professor and Head, Department of Pedodontics and Preventive Dentistry, Kalinga Institute of Dental Sciences, KIIT University, Bhubaneswar Odisha, India

**Keywords:** Oral habits, Preschool children, Prevalence.

## Abstract

**Aim:**

Oral habits during and beyond preschool age are one of the important etiological factors in developing malocclusion and other ill effects on orofacial structures. The objective of the present study was to know the prevalence of deleterious oral habits among 3- to 5-year-old preschool children in Bhubaneswar, Odisha, India.

**Materials and methods:**

This cross-sectional study was conducted among preschool children, in the age group of 3 to 5 years in the city of Bhubaneswar, Odisha, India. To carry out this study, six private schools, two from each of the three electoral constituency, were selected using cluster sampling technique. A total of 500 students, studying in LKG and UKG and their respective mothers/caregivers were selected for the study as per the inclusion/exclusion criteria. Prevalence of different oral habits in children was calculated from the data obtained. Using Statistical Package for the Social Sciences (SPSS), version 17.0 software, Chi-square test was applied to compare the differences present between boys and girls and their significant values (p < 0.05).

**Results:**

The result of this study showed a high prevalence of oral habits (36%) among preschool children in Bhubaneswar, Odisha, India. Lip biting was found to be the most prevalent habit (13.4%), followed closely by thumb sucking (12.8%), bruxism (12.8%), and mouth breathing (11%).

**Conclusion:**

The study revealed a great dearth of a well-established dental education program for preschool children as well as their parents, caretakers, teachers, and pediatricians in order to provide an effective and timely care to the children.

**How to cite this article:** Dhull KS, Verma T, Dutta B. Prevalence of Deleterious Oral Habits among 3- to 5-year-old Preschool Children in Bhubaneswar, Odisha, India. Int J Clin Pediatr Dent 2018;11(3):210-213.

## INTRODUCTION

Habits are acquired automatisms, represented by an altered pattern of muscle contraction with complex characteristics, which proceed unconsciously and on a regular basis.^1^ Repetitive behavior of habits is common in infantile period and most of them are started and stopped spontaneously. One of the most common and earliest repetitive behaviors seen in infantile period is digit sucking.^2^ The reflex of sucking appears in intrauterine life, around 29 weeks,^3^ and disappears during normal growth between the ages 1 and 3% years. The development of habits is considered as a part of the normal sequence of maturation process in children but can have the potential to become a problem or harmful one, under the circumstances of physical, mental, and socioeconomic stress.

An oral habit in infancy and early childhood is normal, and it is considered abnormal over 3 years of age.^3^ Oral habits could be functional or parafunctional. Functional habits result from repeating a normal function, such as nasal breathing, chewing, phonoarticulation, and swallowing, while the parafunctional habits are acquired by practicing a nonfunctional or unnecessary action, such as thumb or lip sucking, bruxism, mouth breathing, and tongue thrusting.^4,5^ The persistence of deleterious parafunctional oral habits have little effect on child health, but play a significant role in altering the position of the teeth, the inter-arch relationship, interfering with the normal growth of the jaws, and the function of the orofacial musculature.^6,7^

Review of literature on oral habits among children revealed wide range of prevalences existing between population, races, and countries,^7,8^ and it is believed to be influenced by various factors, such as gender, rank of the child in the family, feeding methods, socioeconomic status, maternal age, maternal occupation, and education.^8^ Moreover, it is observed that there has been an ascending trend in the prevalence of oral habits in children, in recent times, probably influenced by change in the family and social environment.^8^ The relative prevalence of oral habits in mixed dentition in India has been reported to be as low as 3% in North India and 30% in South India.^9,10^ However, there is paucity of data regarding the prevalence of oral habits among preschool children in India. Hence, the present study was designed to identify the prevalence of deleterious oral habits among 3- to 5-year-old preschool children in Bhubaneswar, Odisha, India.

## MATERIALS AND METHODS

### Source of Data

This cross-sectional study was conducted among preschool children in the age group of 3 to 5 years, in the city of Bhubaneswar, Odisha, India. The research proposal was approved by the Institutional Ethics Committee of Kalinga Institute of Dental Sciences, KIIT University, Bhubaneswar. To carry out this study, six private schools, two from each of the three electoral constituencies, were selected using cluster sampling technique. A total of 549 students studying in LKG and UKG and their respective mothers/caregivers were enrolled in the study.

### Study Design

Prior to the commencement of the study, a notice signed by the principals of the respective schools was sent to the parents through their wards, mentioning the child’s oral habit information collection camp to be held in the schools, on parent/teacher meeting day, and hence, attendance was made compulsory. On the specified day, a self-designed questionnaire pro forma constituting questions with multiple choices on demographic profile, feeding habits, about the child’s past or present oral habits (such as thumb sucking, mouth breathing, lip biting, and bruxism) was distributed among mothers. They were also explained regarding the purpose of the study and honesty in imparting information in the pro forma sheet. Proformas were then collected and checked for the completeness in answering. Mothers/caregivers who were absent for the meeting also received the pro forma at home and returned back to school, which were collected by the investigator later.

### Clinical Examination

All the proformas were screened and a list was prepared consisting of those children whose mothers/caregivers had given a positive response toward presence of deleterious oral habit as mentioned earlier. In a separate appointment, those enlisted children were examined to confirm the presence of deleterious oral habit using following tests:

**Table 1:** **Table 1:** Total and individual prevalence of oral habits in 3- to 5-year-old children

*Type of oral habit*		*Boys (n = 273)*		*Girls (n = 227)*		*Total (n = 500)*		*Chi-square*		*p-value*	
Total (one or more than one habits)		110 (40.3%)		71 (31.3%)		181 (36%)		3.98		0.046*	
Thumb sucking		37 (13.5%)		27 (11.9%)		64 (12.8%)		0.18		0.671	
Mouth breathing		28 (10.3%)		27 (11.9%)		55 (11%)		0.19		0.662	
Lip biting		43 (15.7%)		24 (10.6%)		67 (13.4%)		2.44		0.118	
Bruxism		39 (14.3%)		25 (11.1%)		64 (12.8%)		0.91		0.340	

*Significant (d < 0.05)

- Thumb sucking was evaluated by examining child’s mouth and proclination of maxillary incisors.
- Mouth breathing was confirmed by Mirror test/Fog test, presence of incompetent upper lip combined with inflamed marginal gingiva.
- For lip biting habit bite marks or inflammation of lower lip was observed.
- Bruxism was confirmed by inspecting worn-off cusp tips or incisal edges.

### Statistical Analysis

Prevalence of different oral habits in children was calculated from the data obtained. Using SPSS version 17.0 software, Chi-square test was applied to compare the differences present between boys and girls and their significant values (p < 0.05).

## RESULTS

Out of 549 preschool children, 500 were included in the study for statistical analysis and the rest 49 students were excluded from the study because of the following reasons: (1) Children with gross destruction/extraction of anterior teeth due to caries; (2) children with known systemic respiratory or neuromuscular disorders.

Table 1 depicts the overall and individual prevalence of deleterious oral habits of 3- to 5-year-old children with gender differences.

Overall prevalence of deleterious oral habits (one or more than one in a single child) in 3- to 5-year-old preschool children was found to be 36%. In boys, it was higher (40.3%) compared with girls (31.3%), which was statistically significant (p < 0.05). However, this significant gender-wise differences were not present when individual oral habits were assessed.

## DISCUSSION

One of the contributory factors in the establishment of improper occlusion is child’s deleterious oral habits. Development of these parafunctional habits in the preschool age children is considered as a sign of distress and emotional instability.^11^ Children in particular practice these anomalous habits as a way to attract attention, possibly because they find themselves in a violent family environment, lack of parental attention, lack of emotional maturity, or constant changes in the family.^12^ Besides, poor physical health and chronic illness during infancy can also predispose to the development of these habits. Many authors in most of the studies mentioned that oral habits, if persisted beyond preschool age, play a significant role in the development of dental anomalies and malocclusion as they produce a disequilibrium between the intra- and extraoral muscular activities.^13-15^

Owing to higher prevalence of oral habits observed in the age group of 3 to 5 years in the literature, this age group was selected in the present study.^12,16-18^ The criterion of exclusion of children having lost their anterior teeth was also considered as loss of those teeth would not give a clear picture of influence of oral habits on dentition and orofacial structures.

The overall prevalence of oral habits in this study was found to be 36%, which is in accordance with the studies reported by Rajchanovska and Zafirova-Ivanovska^19^ (35.9%) and Omer and Abuaffan^18^ (30%), and lower than the results reported by Murrieta-Pruneda et al^12^ and Mur-rieta et al^20^ among Mexican preschool children. Contrary to this, Onyeaso and Sote^21^ reported a low prevalence of oral habits (13.14%) among Nigerian preschool children. This wide range of the prevalence of oral habits may be partially accounted to the fact that different oral habits were assessed at different age groups and different methodologies have been used. Nevertheless, the role of cultural and environmental factors on the occurrence of oral habits cannot be ignored.

Further, analysis of data revealed that majority of children had only one oral habit present; however, 69 (38%) children presented with more than one habit. Lip biting was found to be the most prevalent habit (13.4%), which is higher than the observations reported in other studies.^10,12,20^ Both thumb sucking and bruxism were the second most prevalent habit with the incidence rate of 12.8% each. Similar prevalence of thumb sucking habit was reported by Santos et al^16^ and Omer and Abuaffan^18^ among Brazilian and Sudanese preschool children respectively. Conversely, low prevalence of thumb sucking has been reported by many authors.^7,8,10,12,22^ The reported prevalence rate of bruxism in our study (12.8%) was found to be much higher than those reported by Shetty and Munshi^10^ and Murrieta et al.^20^

Mouth breathing (11%) was the least prevalent habit in comparison to other habits and is in accordance with the study conducted by Ahmed and Abuaffian;^23^ however, a slightly higher percentage of prevalence was reported among Indian and Albanese school children respectively.^24,25^

In all the oral habits assessed, boys had higher prevalence than girls; however, these differences were not significant when individual habits were compared. Conversely, a significant value was obtained (p < 0.05) when overall prevalence of oral habits was compared (41.3 *vs* 31.3%). Similar gender-wise difference in the occurrence of oral habits was reported by Santos et al.^16^ A higher level of expectation in the performances of boys by the family and nonacceptance/rejection by the peer group in the society could be attributed to this finding.

This study also revealed a great dearth of a well-established dental education program for preschool children as well as their parents, caretakers, teachers, and pediatricians in order to provide an effective and timely care to the children.

## CONCLUSION

Oral habits, if they persist beyond the preschool age, have detrimental effects on the developing dentition, oral functions, and facial esthetics. The present study provides an insight into the various aspects of oral habits, like their prevalence and implications on primary dentition and facial esthetics. The results of this study also warrant the need for educating the children, parents as well as teachers about the deleterious effects produced by such habits on the development of normal occlusion and importance of timely intervention.
